# Dynamic Role of Phospholipases A2 in Health and Diseases in the Central Nervous System

**DOI:** 10.3390/cells10112963

**Published:** 2021-10-30

**Authors:** Grace Y. Sun, Xue Geng, Tao Teng, Bo Yang, Michael K. Appenteng, C. Michael Greenlief, James C. Lee

**Affiliations:** 1 Department of Biochemistry, University of Missouri, Columbia, MO 65211, USA; SunG@missouri.edu; 2 Department of Biomedical Engineering, University of Illinois at Chicago, Chicago, IL 60607, USA; xgeng6@uic.edu (X.G.); taoteng@uic.edu (T.T.); 3 Chemistry Department, University of Missouri, Columbia, MO 65211, USA; boyang@fas.harvard.edu (B.Y.); mkappenteng@mail.missouri.edu (M.K.A.); GreenliefM@missouri.edu (C.M.G.)

**Keywords:** phospholipases A2, central nervous system, lysophospholipids, oxidized fatty acids, oxylipins, stroke, Alzheimer’s disease

## Abstract

Phospholipids are major components in the lipid bilayer of cell membranes. These molecules are comprised of two acyl or alkyl groups and different phospho-base groups linked to the glycerol backbone. Over the years, substantial interest has focused on metabolism of phospholipids by phospholipases and the role of their metabolic products in mediating cell functions. The high levels of polyunsaturated fatty acids (PUFA) in the central nervous system (CNS) have led to studies centered on phospholipases A2 (PLA2s), enzymes responsible for cleaving the acyl groups at the *sn*-2 position of the phospholipids and resulting in production of PUFA and lysophospholipids. Among the many subtypes of PLA2s, studies have centered on three major types of PLA2s, namely, the calcium-dependent cytosolic cPLA2, the calcium-independent iPLA2 and the secretory sPLA2. These PLA2s are different in their molecular structures, cellular localization and, thus, production of lipid mediators with diverse functions. In the past, studies on specific role of PLA2 on cells in the CNS are limited, partly because of the complex cellular make-up of the nervous tissue. However, understanding of the molecular actions of these PLA2s have improved with recent advances in techniques for separation and isolation of specific cell types in the brain tissue as well as development of sensitive molecular tools for analyses of proteins and lipids. A major goal here is to summarize recent studies on the characteristics and dynamic roles of the three major types of PLA2s and their oxidative products towards brain health and neurological disorders.

## 1. Introduction

**Figure 1:**
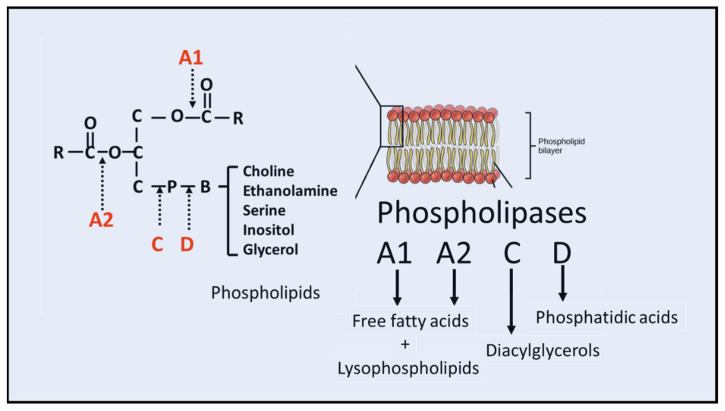
Phospholipases acting on phospholipids. Action of phospholipases A1, A2, C and D on phospholipids resulting in free fatty acids and lysophospholipids, diacylglycerol and phosphatidic acids, respectively.

Phospholipids are the major component of the membrane lipid bilayer in mammalian cells. These molecules are comprised of two acyl or alkenyl chains, a glycerol backbone and several different phospho-base groups (ethanolamine, choline, serine and inositol). Membrane phospholipids are substrates of phospholipases A1, A2, C and D, enzymes producing small molecules that serve as mediators with important functional roles in cell metabolism (Figure 1). While phospholipases A1 (PLA1s) and PLA2s are responsible for cleavage of the acyl groups at the *sn*-1 and *sn*-2 positions of glycerophospholipids, respectively, their actions also result in the production of lysophospholipids (phospholipids with one acyl chain), molecules known to possess detergent-like properties. PLA2s are comprised of a large group of superfamily (more than 50 subtypes) based on their calcium dependence and cellular localization. These enzymes not only play important role in remodeling cell membrane structure and homeostasis, but also engage in different aspects of cell metabolism through specific signaling pathways [1,2]. Over the years, substantial interest has focused on PLA2s because acyl groups at the *sn*-2 position of glycerophospholipids are largely polyunsaturated and serve as substrates for oxygenases which form eicosanoids and docosanoids, lipid mediators that play important roles in regulating cell immune functions and inflammatory response.

**Figure 2:**
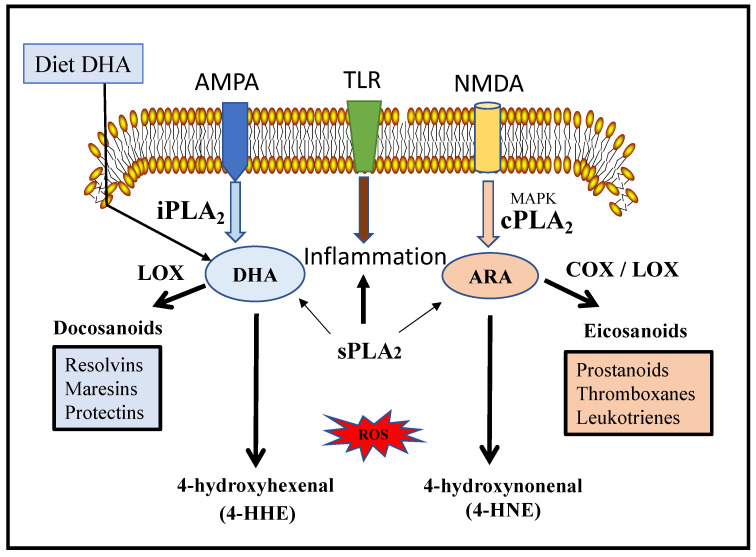
Receptor- signaling pathways for iPLA2, sPLA2 and cPLA2 leading to production of docosahexaenoic acid (DHA) and arachidonic acid (ARE). In turn, DHA and ARA are metabolized by cyclooxygenases (COX) and lipoxygenases (LOX) to produce docosanoids and eicosanoids and undergo peroxidation to produce 4-hydroxyhexenal (4-HHE) and 4-hydroxynonenal (4-HNE), respectively. Abbreviations: AMPA, α-amino-3-hydroxy-5-methyl-4-isoxazole proprionic acid receptor, TLR, Toll-like receptors, NMDA, N-methyl-D-aspartate receptor.

Phospholipids in the central nervous system (CNS) are enriched in arachidonic acid (ARA, 20:4n-6) and docosahexaenoic acid (DHA, 22:6n-3). These PUFAs are released from phospholipids through activities of three major types of PLA2s, namely, the calcium-dependent cytosolic cPLA2, the calcium independent iPLA2 and the calcium-dependent secretory sPLA2 (Figure 2). Each type of PLA2 has specific molecular structures, modes of actions and depending on the cell types, they have different calcium requirements and association with different receptor-mediated signaling pathways [3]. Along with discoveries of different subtypes of PLA2, studies on their biological functions were advanced by development of specific inhibitors [4]. However, despite of abundant studies linking PLA2s to immune cells and inflammatory diseases in the peripheral system, studies on specific PLA2 in the CNS are relatively limited due to the complex makeup of different cell types in different brain regions. Previous reviews from our laboratories had been confined mainly to cPLA2 [5,6]. In more recent years, improved techniques for cell type separation and advanced lipidomics for analysis of phospholipids have provided new information on genetic variances of different PLA2s, their underlying mechanism(s) of action and different profiles of oxidative products (oxylipins). A major goal of this review is to summarize recent studies on the dynamic role of the three major subtypes of PLA2s and their metabolic products, including ARA, DHA and lysophospholipids, in health and disease in the CNS. Because of the voluminous amount of information, the authors regret lacking emphasis of earlier studies related to genetic composition and mechanisms of action of protein domains for these PLA2.

## 2. Structure and Function of cPLA2

Cytosolic phospholipases A2 (cPLA2s) are intracellular enzymes with 749 amino acids and a molecular weight around 85 kDa. The cPLA2 family is comprised of six isoforms, namely, cPLA2α, -β, -γ, -δ, -ε and -ζ [1]. Except for cPLA2γ, which lacks the C-domain, other cPLA2s subgroups are characterized by the presence of a highly conserved C2 domain for binding Ca^2+^ and the C-terminal domain containing the catalytic active site using the Ser-Asp dyad for catalysis [7]. The catalytic domain also contains a number of serine residues and Ser505, 515 and 727 are susceptible for phosphorylation by a number of protein kinases including the Mitogen-activated protein kinases (MAPKs), Ca^2+^/calmodulin-dependent protein kinase II (CAMKII) and mitogen-activated protein interacting kinases (MNK1) [8]. Phosphorylation of cPLA2 increases phospholipid binding at low calcium concentration and facilitates translocation of enzyme in the cytoplasm to different intracellular organelles including the nuclear, mitochondrial, lysosomal and the plasma membranes [9]. cPLA2 also interacts with other lipid molecules, such as ceremide-1-phosphate and phosphatidylinositol bisphosphate (PIP2), although how these lipids modulate enzyme activity remains to be further investigated [8]. More recently, lactosylceramide (LacCer) has been found to bind cPLA2 in the C-2 domain and in CHO-W11A cells, LacCer could trigger cPLA2 activity resulting in the release of ARA [10]. In a study with astrocytes, increase in cPLA2 activity due to LacCer was linked to promotion of inflammation and neurodegeneration in the mouse model of experimental autoimmune encephalomyelitis (EAE) [11]. Interestingly, besides binding with cPLA2, LacCer can also interact with NADPH oxidase to generate reactive oxygen species (ROS) and subsequently engage in inflammatory diseases such as atherosclerosis and cancer [12].

Among many subtypes of cPLA2s, cPLA2α is probably the most studied. This cPLA2 appears to be present ubiquitously in all brain cells, including neurons, astrocytes and microglia [5]. In an in vitro study with primary neurons, stimulation of neurons with the excitatory glutamate receptor could result in rapid activation of the Ras/Raf/MEK/ERK pathway and subsequently phosphorylation of cPLA2 [13]. In microglial cells, stimulation of the Toll-like receptor with lipopolysaccharide (LPS) could induce ERK1/2-dependent cPLA2 phosphorylation and upregulation of the inflammatory pathway involving NF-kB [14,15]. A more recent study using quantitative proteomics also identifies upregulation of NF-kB associated inflammatory proteins upon treatment of microglial cells with LPS [16]. Taken together, these studies demonstrate the role of protein kinases for phosphorylation of cPLA2 in neuronal excitation and glial inflammation.

Substantial evidence has indicated cPLA2 to preferably target phosphatidylcholine (PC) and release ARA and lysophosphatidylcholine (LPC) [17]. ARA is a key substrate of cyclooxygenases (COX), lipoxygenases (LOX) and cytochrome P450 and depending on the cell type, these reactions result in synthesis of oxygenated products such as prostaglandins, leukotrienes and thromboxane B2 (Figure 2). Except for lipoxin, these ARA-derived lipid mediators are known to mediate inflammatory responses through acting on receptors in a cell-specific manner. Studies to elucidate the role of cPLA2 on neuro-inflammatory events include using genetic deletion, antisense oligonucleotides and cPLA2 specific inhibitors [18,19,20,21,22]. In studies using cell and animal models, arachidonyl trifluoromethyl ketone (AACOCF3 or ATK) has been successfully used to suppress cPLA2 activity and production of eicosanoids [17,23,24]. AACOCF3 also mitigates cPLA2-induced pathology in cardiovascular disease in mouse model, in human aortic smooth muscle cells [25] and in type II alveolar epithelial cells exposed to cigarette smoke condensate [26].

## 3. cPLA2 in Cerebral Ischemia, Spinal Cord and Traumatic Brain Injury

### 3.1. Cerebral Ischemia

Early studies have implicated the role of PLA2 in the rapid release of PUFA in brain due to different forms of brain injuries [27]. Studies with animal models have demonstrated increases in oxidative stress, neuronal excitation and glial cell activation, which are factors contributing to stimulation of cPLA2 in cerebral ischemia. However, due to the complex cellular makeup of the brain tissue, studies to examine the role of PLA2 in neurons, astrocytes and microglial in animal models of ischemic stroke have not been forthcoming. Under the ischemic condition, PLA2-induced release of PUFA is exacerbated by the lack of oxygen supply and decrease in ATP required for conversion of the fatty acids to their acyl-CoA. Consequently, perturbation of the deacylation-reacylation cycle mediated by phospholipases A2 and acyltransferases is an important factor for alterations of cellular phospholipids and PUFA during cerebral ischemia [1,28]. Interestingly, stroke-mediated increase in PUFA in brain can also be observed in plasma, thus making analysis of PUFA in plasma a useful marker for assessing the extent of brain damage in stroke patients [29].

### 3.2. Traumatic Brain Injury (TBI)

Stimulation of cPLA2 and the resulted release of ARA and lipid mediators are important factors leading to inflammation and pain in TBI. In a mouse cortical impact model, administration of AACOCF3, the cPLA2 inhibitor, could mitigate impaired autophagy that led to neuronal cell death [9]. AK106-001616, another selective inhibitor of cPLA2 could reduce the prostaglandin E2 (PGE2) and leukotriene B4 (LTB4) production in cells and in models of inflammation, neuropathic pain and pulmonary fibrosis [30]. These studies demonstrated the important role of cPLA2 in inflammatory events associated with TBI. At a subcellular level, cPLA2 was found to play a role in TBI-induced changes in lysosomal membrane permeability, leading to subsequent neuronal impairment [9]. In this study, increase in phosphorylation of cPLA2 was detected as early as 1 h after TBI and analysis of lysosomal membranes indicated decreases in phospholipids with PUFA, such as phosphatidylcholine (PC) 18:0/20:4, PC 18:0/22:6, phosphatidylethanolamine (PE) 16:0/22:6 and PE-p 18:0/22:6, 16:0/22:6 and 18:0/20:4 and increases in lysophosphatidylcholine (LPC) 16:0, 18:0 and lysophosphatidylethanolamine (LPE) 18:0. Similarly, administration of arachidonyl trifluoromethyl ketone (AACOCF_3_) could normalize changes in lysosomal membrane lipids and attenuate neuronal cell death [9].

### 3.3. Spinal Cord Injury (SCI)

SCI is also associated with increase oxidative stress, neuroinflammation, glial activation and lipid peroxidation. In a study with a rat spinal cord contusion model, rapid elevation of p-cPLA2 was observed 2 h after injury and the increase in cPLA2 protein expression remained prominent up to 7 days after SCI [31]. In this SCI model, cPLA2 was shown to play an important role in the secondary injury cascade and inhibition of cPLA2 at 30 min postinjury could ameliorate SCI-induced motor deficits and reduce cell loss and tissue damage [32].

## 4. cPLA2 in Other Neurodegenerative Diseases

### Alzheimer’s Disease (AD)

Alzheimer’s disease (AD) is a major neurodegenerative disease marked by the deposition of amyloid plaques and neurofibrillary tangles in the brain. A number of studies have implicated the role of cPLA2 in release of ARA and increase in oxidative/nitrosative pathways in AD [33,34,35]. The increasing focus on PLA2 is due to a link with their products to synaptic involvement in learning and memory [36,37,38]. In a recent study with the 5xFAD transgenic mice, changes in lipid species, such as an increase in lysophospholipids in the hippocampus, provided support to the increase in PLA2 activity in AD brain [39]. In a more recent study with the APP knock-in (App^N-G-F/N-G-F^) mice, there was evidence for a more aggressive Aβ accumulation, reactive gliosis and cognitive deficits as compared to other transgenic mouse models [40,41]. The APP knock-in mice also showed increase in cPLA2 levels in the brain [42]. A study using an unbiased lipidomic approach and biochemical assessments in different AD mouse models indicated changes in the Land’s cycle activity, such as an age-dependent increase in lysophosphatidylcholine level and cPLA2 activity and changes in phospholipids were marked by a progressive decline in behavior and memory [43]. However, since there are many PLA2 subtypes in different brain cells, more studies are needed to better define the lipid changes in a subcellular and subregion level. In a study with aged (16-month-old) transgenic Tg2576 AD mice, a subgroup (20%) of mice showed resilience to the spatial memory test. Genetic analysis of this subgroup unveiled the possible role of PLA2G4E (cPLAɛ) associated with the resilience to the spatial memory [44]. This finding may have important implication as analysis of AD patients also showed a defect in this cPLA2ɛ gene. Moreover, in a study with the APP/PS1 mice, administration of adeno-associated viral vector of the cPLA2ɛ gene to hippocampal neurons could restore the cognitive defects in these mice [44].

There is growing evidence that amyloid beta peptide (Aβ) released from amyloid precursor protein (APP) can become cytotoxic upon aggregation to oligomeric form [45]. Our earlier studies with astrocytes demonstrated the ability of oligomeric amyloid beta (oAβ) to activate cPLA2 and alter membrane physical properties [46] and mitochondrial function [47]. Aggregated Aβ_1-42_ was shown to stimulate cPLA2 phosphorylation in cortical neurons and regulation memory deficits and neuronal apoptotic cell death [13,38,48,49,50]. Studies further showed the ability of oAβ to activate inflammatory pathways in microglia and in turn, cPLA2 played a role in microglial functions including uptake of amyloid beta [51,52]. In these studies, antisense oligonucleotide against cPLA2 could abrogate the oxidative events and neuronal damage [49]. In addition, both azelnidipine (ALP), a dihydropyridine calcium channel blocker known for its treatment of hypertension and methylarachidonyl fluorophosphate (MAFP), a cPLA2 inhibitor, also suppressed oAβ-induced translocation of the nuclear factor kappa-light-chain-enhancer of activated B cells (NFκB) p65 subunit to nuclei in cerebral endothelial cells, suggesting that cPLA_2_ activation and calcium influx are essential for oAβ-induced NFκB inflammation [53].

Since apolipoprotein E4 (APOE4) has been identified as a prevalent genetic risk factor driving the development of AD, recent studies have attempted to link lipid peroxidation products such as 4-hydroxynonal (HNE) which can bind with different APOE cysteine residues [54]. There is evidence for higher levels of cPLA2 expression in AD patients carrying APOE 3/4 as compared to APOE 3/3 genotype [55]. In this latter study, astrocytes isolated from mice transfected with human APOE4 also showed greater levels of inflammatory markers, such as reactive oxygen species (ROS) and inducible nitric oxide synthase (iNOS), as compared with mice transfected with APOE3 [55]. Furthermore, inhibition of cPLA2 could abrogate the increase in inflammation in APOE4-TR mice. Taken together, these results suggest the important role of cPLA2 in exerting greater inflammatory activities in brain of APOE4 genotype. Expectedly, more studies are needed to examine underlying mechanism(s) whereby cPLA2 and its downstream products may play a role in driving the AD pathology, especially those associated with the E4 allele.

## 5. Structure and Function of iPLA2

Group VI (GVI) phospholipases A2, also known as iPLA2, belong to the group of calcium-independent phospholipases A2. Currently, six subgroups of iPLA2 have been identified: GVIA (iPLA2β; PNPLA9), GVIB (iPLA2γ; PNPLA8), GVIC (iPLA2δ; PNPLA6), GVID (iPLA2ε; PNPLA3), GVIE (iPLA2ζ; PNPLA2) and GVIF (iPLA2η; PNPLA4) [56]. Molecular weights of these subgroups of iPLA2 range from 84 to 91 kDa, depending on the cellular localization. All GVI PLA2s have a patatin-like lipase domain and share a GXSXG catalytic motif together with multiple strings of ankyrin motif [2,57]. Among the subgroups, GVIA PLA2 (iPLA2β) and GVIB PLA2 (iPLA2γ) are the most studied [2].

### 5.1. GVIA PLA2 (iPLA2β)

GVIA PLA2 (iPLA2β) is an 85 kDa protein with 752 amino acids [56]. This enzyme is widely present in the peripheral organs as well as in the CNS and is thus regarded as the most prominent phospholipase playing a house-keeping role for maintaining membrane homeostasis [58]. In addition, GVIA PLA2 also plays a role in cell proliferation, cell death and signal transduction and thus posts impact on diseases including cancer, cardiovascular abnormalities, glaucoma, periodontitis and nerve degeneration [56,59]. In recent years, deficiency in iPLA2β has been reported to elevate mitochondrial lipid peroxidation, resulting in mitochondrial dysfunction [60]. Mutation of this PLA2 also results in reduction of mitochondrial potential, leading to attenuation of calcium uptake and calcium retention capacity in mitochondria [61]. Studies on iPLA2 have been aided by the specific inhibitor, bromoenol lactone (BEL). In hippocampal pyramidal neurons, inhibition of iPLA2 by BEL increases amplitude of the AMPA receptor activity [62].

Studies on iPLA2β indicate that this is the major phospholipase responsible for the release of DHA from glycerophospholipids [63,64,65]. In an earlier study, iPLA2β^−/−^ and iPLA2β^+/+^ mice infused intravenously with [1-^14^C]-DHA demonstrated a significant decrease in baseline activity for uptake of labeled DHA by the iPLA2β^−/−^ mice [64]. Studies with iPLA2β KO mice also showed a decrease rate of incorporation of unesterified DHA from plasma into brain phospholipids and, thus, resulted in alterations of the brain fatty acids and lysophospholipids in the knockout (KO) mouse brain [66]. Interestingly, depletion of iPLA2β is marked by an increased in mRNA and activity of cPLA2α, suggesting presence of a compensatory metabolic link between these two types of PLA2s. iPLA2β KO mice show minimal neuropathology during birth but with increasing age, there is evidence for motor disturbances, cerebellar neuron loss and striatal α-synuclein accumulation. In addition, the aged iPLA2β KO mice also exhibit activations of microglia and astrocytes and increase in tumor necrosis factor α (TNFα) and iNOS) production, suggesting the role of this PLA2 in mediating oxidative and inflammatory events with increasing age [63].

### 5.2. iPLA2γ

iPLA2γ is a membrane-bound enzyme with 782 amino acids and molecular weight of 90 kDa. This enzyme has the lipase consensus sequence Gly-X-Ser-X-Gly in the C terminal and a Ser-Asp dyad active site in the catalytic domain [67]. iPLA2γ also shows a motif for mitochondrial localization in the N-terminal and a nucleotide binding motive together with a peroxisome localization signal at the C terminal [67]. While the S-enantiomer of BEL is known to preferably inhibit iPLA2β, iPLA2γ is preferably inhibited by the R-enantiomer. Interestingly, there is evidence that iPLA2γ can interact with both diacyl and alkenyl acyl form of phosphatidylcholine (PC) and phosphatidylethanolamine (PE) as well as hydrolysis of fatty acids in either *sn*-1 or *sn*-2 position [67]. In agreement with the mitochondrial localization, iPLA2γ KO mice show aberrant mitochondria structure with swelling and abnormal cristae in the hippocampus. Genetic deletion of iPLA2γ also shows decrease in cardiolipin (CL), the specific phospholipid in mitochondrial membrane. There is further evidence for iPLA2γ to target oxidized CL [68,69]. Some of these activities may be due to the putative serine residues in this molecule, rendering it susceptible to phosphorylation by protein kinases including protein kinase C (PKC) and extracellular signal-regulated kinase (ERK) [70,71]. Obviously, more studies are needed to better understand the role of these protein kinases in mediating activity of the enzyme under different conditions. iPLA2γ deficiency is shown to enhance α-amino-3-hydroxy-5-methyl-4-isoxazole proprionic acid (AMPA) receptor destabilization and tau phosphorylation [72] and AMPA receptor overstimulation in hippocampus may play an important role in neuron cell death [73].

## 6. iPLA2 and Neurological Diseases

Mutations of GVI PLA2 gene have been shown to cause a number of neurological abnormalities including infantile neuroaxonal dystrophy (INAD), neurodegeneration with brain iron accumulation (NBIA), autosomal recessive early-onset dystonia-Parkinson disease with Lewy body pathology and accumulation of hyperphosphorylated tau [57,60,63,74]. In a study with Drosophila, KO of the iPLA2β gene results in reduced survival, locomotor deficits, hypersensitivity to oxidative stress and increase in lipid peroxidation [60]. Deficiency of iPLA2-VIA in Drosophila also exhibits neurotransmission defects and degeneration of DA neurons [75]. In this last study, iPLA2-VIA loss is linked to the disturbance of membrane lipids leading to endoplasmic reticulum stress.

## 7. Structure and Function of sPLA2

sPLA2s are small molecular weight proteins (20–40 kDa) present in different mammalian organs including the CNS. This group of enzymes are transcriptionally induced in cells upon challenge by toxins and pro-inflammatory cytokines and under infectious conditions such as arthritis and sepsis. Upon transcription, active sPLA2 are subsequently secreted and thus can interact with other cells in the extracellular milieu. Under these conditions, sPLA2 are often induced together with the C-reactive protein and serve as markers of inflammation [76]. Studies have identified more than 10 isoforms of sPLA2 e.g., IB, IIA, IIC, IID, IIE, IIF, III, V, X and XIIA, which are distributed among different cell types and body organs [77]. The ribbon model of human group III sPLA2 showed three helices, one calcium binding loop, five disulfide bonds and His34 and Asp63 in the active site [77]. In the extracellular milieu, sPLA2s require high levels of calcium for activation [78]. Although sPLA2s do not seem to have much substrate specificity, their ability to release ARA can contribute to the pool of inflammatory lipid mediators similar to those produced by cPLA2 [79].

Previous studies on different groups of sPLA2 have focused mainly on their specific functions in the peripheral system. Among these isoforms, sPLA2-IIA is probably the most studied due to its implication in atherosclerosis, septic shock, peritonitis, rheumatoid arthritis and other host defense mechanisms [80]. In studies with cells, increase in sPLA2-IIA expression is shown in response to inflammatory stimuli, such as IL-1β, IL-6, TNFα in association with the NF-kB and signal transducer and activator of transcription 3 (STAT3) transcription pathways. There are examples of other sPLA2 subtypes playing roles in specific physiological events in the body. For example, group IIF is associated with epidermal hyperplasia, IIE in regulating hair follicle homeostasis and IID in suppression of lymphoid immune cells [81]. Since sPLA2-IIF is more hydrophobic than other sPLA2s, it can better penetrate membranes and disrupt lipid monolayers and bilayers [82]. Together, different sPLA2s have been implicated to play important roles in mediating different body functions including epidermal hyperplasia, male reproduction, anaphylaxis colonic diseases and atherosclerosis.

A major difficulty to delineate the role of PLA2 in specific cell types in the brain is the lack of protocols/methods to isolate these cells. As a result, many studies have reverted to using immortalized cell lines. A study by Sheng et al. examined the effects of proinflammatory cytokines and LPS on induction of iNOS and sPLA2-IIA in primary and immortalized astrocytes and microglial cells [83]. In this study, proinflammatory cytokines (TNFα, IL-1β and interferon-γ (IFNγ) and LPS could upregulate sPLA2-IIA mRNA and protein in primary and immortalized rat astrocytes (DITNC) but not in primary or immortalized microglial cells [83]. In contrary, expression and secretion of sPLA2-IIA are observed in human microglia-like promonocytic THP-1 cells and human primary astrocytes with proinflammatory cytokines [84]. Obviously, more studies are needed to elucidate the conditions to stimulate sPLA2-IIA in different cell types in the CNS.

Studies on sPLA2-IIA in mice are also hampered due to lacking this gene in many mouse strains [85]. However, this problem is ratified by transfecting the human sPLA2-IIA gene into these mice. Interestingly, mice harboring the human sPLA2-IIA exhibit epidermal pathology [86] and increase in atherogenesis [87]. In a study, an attempt was made to analyze the eicosanoids and metabolic mediators in the cPLA2α^−/−^ mice [88] and transgenic sPLA2-IIA^TGN^ (both on C57BL/6J background) to determine the metabolites from these PLA2 in the development of arthritis [79]. Results indicated no distinct separations and thus both cPLA2 and sPLA2-IIA could play a role in the production of these metabolites and development of arthritis. It is important to recognize that although this mouse strain lacks sPLA2-IIA, it does not preclude the possible presence of other sPLA2 isoforms which may provide similar physiological functions.

## 8. sPLA2 in Extracellular Vesicles

Early studies have identified proteins and phospholipases (A2, C and D) in bioactive vesicles (exosomes) secreted from cells. Release of these vesicles offer the possibilities to mediate intercellular signaling mechanisms [89]. sPLA2-IIA and its metabolic products are identified in the extracellular vesicles secreted from astrocytes [80]. Expectedly, these vesicles in the extracellular milieu can interact and alter functions of other cells including neurons. Studies with primary human astrocytes showed increase in sPLA2-IIA in response to pro-inflammatory mediators and the secreted sPLA2-IIA present in the conditioned medium could cause toxic effects to SH-SY5Y neuroblastoma cells [84]. sPLA2-IIA also plays an integral role in regulating vascular inflammation and increases risk of cardiovascular diseases (CVD) [90]. In fact, a baseline increase in sPLA2-IIA levels was observed in subjects with early diagnosis of acute respiratory distress syndrome [91]. In the bronchoalveolar lavage fluid, pools of sPLA2-IIA (protein and mRNA) were identified in the extracellular vesicles [91]. In astrocytes activated by cytotoxic Aβ, release of sPLA2-IIA was observed together with activation of calcium signals and treatment with aristolochic acid (sPLA2 inhibitor) could counteract Aβ-induced neurotoxicity [92].

## 9. sPLA2-IIA in Neurodegenerative Diseases

### 9.1. Alzheimer’s Diseases

Despite extensive studies on sPLA2 in infectious and inflammatory diseases, relatively little is known about their role in neurodegenerative diseases. In an early study, autopsy samples from AD patients showed a 4-fold increase in levels of sPLA2-IIA mRNA in AD hippocampi as compared to non-demented elderly brains [93]. Double staining of brain sections also showed a high level of sPLA2-IIA-immunoreactivity in the glial fibrillary acidic protein (GFAP) astrocytes and in the Aβ-containing plaques. In this study, stimulation of cultured astrocytes with Aβ42 and IL-1β resulted in increase in sPLA2-IIA immunoactivity [93]. Furthermore, another study showed that sPLA2-IIA secreted from astrocytes could regulate APP processing in neuronal cells [94]. Taken together, these studies demonstrated a clear correlation between sPLA2-IIA in reactive astrocytes around the amyloid plagues in AD brain.

### 9.2. Cerebral Ischemia

The lack of sPLA2-IIA in many mouse strains has precluded studies to examine its role in cerebral ischemia using the murine model systems. However, in a study using a rat model in which cerebral ischemia was induced by occlusion of the middle cerebral artery (MCAO), measurement of sPLA2-IIA mRNA expression indicated a biphasic increase at 30 min and at 12 h to 14 days [95]. In situ hybridization of sPLA2-IIA mRNA in the brain sections showed an early-phase increase in the affected ischemic cortex and a later phase increase mainly in the penumbral area. Immunohistochemistry and confocal microscopy of the brain sections indicated sPLA2-IIA immunoreactivity associated mainly with reactive astrocytes [95]. Administration of a nanocrystal formulation of PX-18, a sPLA2 inhibitor, exerted neuroprotective effects in a cerebral ischemia/reperfusion model in gerbils [96].

### 9.3. Spinal Cord Injury

A study using a spinal cord injury (SCI) model indicated localization of sPLA2-IIA in oligodendrocytes but not in astrocytes or Schwann cells [97]. In this study, sPLA2-IIA was shown to play a role in mediating oligodendrocyte death after SCI. Quantitative reverse transcription polymerase chain reaction (RT-PCR) analysis indicated upregulation of sPLA2-IIA and IIE mRNA in the rat spinal cord as early as 4 h after injury. Interestingly, besides sPLA2-IIA and IIE, other sPLA2 isoforms such as sPLA2 1B, IIC, V, X and XII, were also present in the spinal cord [97].

Recently, sPLA2 has been implicated in early spinal mechanisms of neuronal excitability and nociception. In a study with a rat nerve root compression model, administration of sPLA2 inhibitor ameliorated mechanical allodynia and attenuated the increase in inflammatory factors (IL-1β, TNFα and IL-1α) due to the injury [98]. These findings agree with the role of sPLA2 regulating neuronal excitability, possibly related to the glutamate signaling and inflammatory cascades. In addition, the increase in inflammatory responses, nerve root compression is also associated with neuropathic pain. Neurochemical and immunohistochemical study identified glial activation together with increases in PLA2 and 8-hydroxyguanosine (8-OHG), a marker of oxidative stress [99]. Treatment with meloxicam (a COX-2 inhibitor) not only reduced activation of astroglial cells, but also prevented neuropathic pain and the oxidative stress in the nerve compression model [99]. To further imply action of sPLA2, thioetheramide-phosphorylcholine, a sPLA2 inhibitor, was shown to attenuate mechanical allodynia and neuronal excitability in this spinal cord injury model [98]. Indeed, loading this sPLA2 inhibitor in phospholipid micelle form proved to be a promising therapeutic target for suppressing neuropathic pain [100].

## 10. PLA2 and Lysophospholipids

In addition to the release of *sn*-2 fatty acids, the enzymatic action of PLA2 also produces lysophospholipids which are known for their detergent-like properties. These molecules can modify properties of the membrane lipid bilayer and be transported effectively across the blood–brain barrier (BBB). In fact, recent studies suggest the use of lysophospholipids as a carrier for fatty acids. Dietary DHA administered in the form of LPC was shown to enhance brain phospholipids with DHA better than with the free fatty acid alone [101,102]. Recent studies indicated transport of DHA-lysophospholipids to brain through a specific Na-dependent transporter “major facilitator superfamily domain containing 2a (Mfsd2A)” expressed in the endothelium of the blood brain barrier (BBB) [103,104]. This transporter is also present in different mammalian species. A cryo-electron microscopic study reveals 12 transmembrane helices and an amphipathic cavity containing the Na^+^ and lysolipid binding site [105]. The important role of this transporter in brain pathology is demonstrated by a recent study showing a genetic variant of this protein to cause congenital microcephaly and hypomyelination [106,107]. Consequently, more studies are needed to understand the physiological role of this transporter for enrichment of DHA in the brain.

Lysophospholipids are substrates for the reacylation process mediated by lysophospholipid acyltransferases and the ATP-dependent activation of acyl-CoA [28]. Considering the role of cPLA2 in the release of ARA under stimulated conditions, activity of this “Land’s cycle” is particularly important in the maintenance of ARA homeostasis in the cell [108]. Due to different types of phospholipids present in the membrane, different isoforms of lysophospholipid acyltransferases are present in the body organs to mediate phospholipid homeostasis [109]. In human monocytes, lysoPC acyltransferase 3 is found to regulate incorporation of ARA into PC [110]. In the brain, an LPI acyltransferase 1 (LPIAT1) is involved in regulating neuronal function. Variants in the membrane-bound O-acyltransferase family member 7 (*MBOAT7*) which encodes LPIAT1 is involved in intellectual disability associated with epilepsy and autism spectrum disorder (ASD) [111]. A recent review by Kita et al. also demonstrated the role of different lysophospholipid acytransferases on mediating specific physiological and pathological functions in the neural and peripheral system [1].

**Figure 3:**
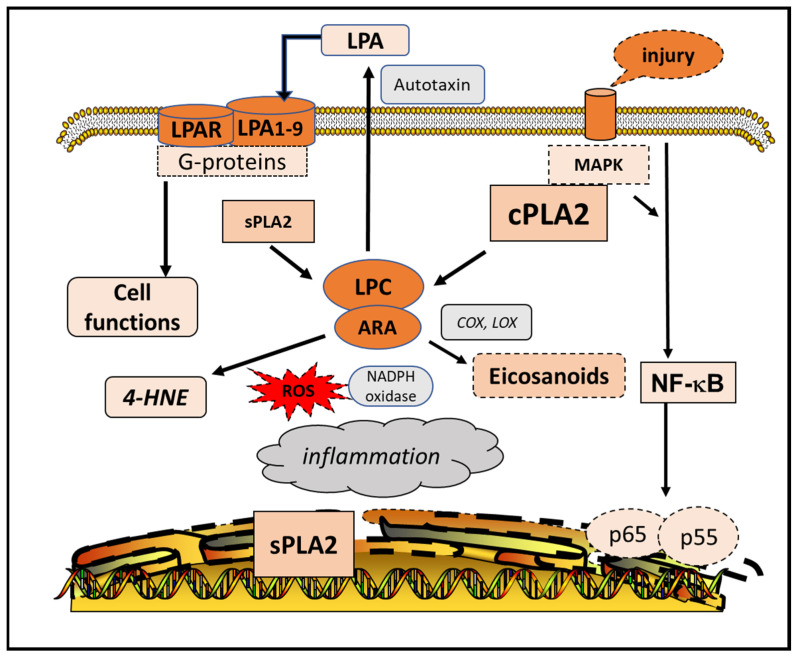
Role of lysophospholipids in metabolic pathways. Cell injury leads to activation of cPLA2 and NF-kB pathway and transcriptional increase in sPLA2. Activation of sPLA2 and cPLA2 result in the release of ARA and lysophosphatidylcholine (LPC). LPC is converted to lysophosphatidic acid (LPA) via the extracellular autotaxin. LPA interacts with G-protein receptors to regulate cell metabolism. Abbreviations: 4-hydroxynonenal (4-HNE), nuclear factor kappa-light-chain-enhancer of activated B cells (NF-kB), reactive oxygen species (ROS), mitogen-activated protein kinases (MAPK).

In brain injury and neuro-inflammatory diseases, increases in LPC have been regarded as a lipid mediator for astrogliosis and other deleterious events associated with neurodegeneration [112]. Recently, substantial interest has been placed on lysophosphatidic acid (LPA), which is regarded to serve as a second messenger for regulating many receptor-mediated cell functions [113]. Although LPA is produced through deacylation of phosphatidic acid (PA) by PLA1 or PLA2 [114,115], there is evidence that this lysophospholipid can also be derived from LPC through autotaxin, a soluble enzyme with PLD-like activity and is present in extracellular fluids such as plasma and cerebrospinal fluid (Figure 3) [116,117]. Recent studies have unveiled the multifunctional role of LPA to act on G-protein-coupled receptors (1–9) (Figure 3) [118,119]. Interestingly, since sphingosine 1-phosphate (SIP) shares a basic structure similar to LPA, both type of lipids can also interact with multiple G-protein-coupled receptors [119]. Activation of these receptors by LPA or SIP stimulates different downstream signaling pathways responsible for regulating intracellular metabolism. Genetic abrasion of LPA receptors leads to aberrant neurodegenerative diseases including AD and neuropathic pain [118,120]. Activation of LPA receptor 1 in macrophages is found to regulate pathology of multiple sclerosis [121]. Furthermore, measurement of LpaR1 expression in blood mononuclear cells is used as a marker for onset/relapse and severity in the EAE model and multiple sclerosis patients. Consequently, future studies to better define the LPA receptors and their signaling pathways should further advance understanding of the pathology of these neurological diseases.

## 11. PLA2-Mediated Production of Oxylipins and Lipid Peroxidation Products

Studies on PLA2s demonstrated a Yin-Yang mechanism for the release of ARA and DHA through the cPLA2 and the iPLA2, respectively (Figure 2) [122]. Both ARA and DHA are subject to enzymatic and non-enzymatic free-radical oxidation reactions resulting in production of oxylipins. Depending on the cell types, these oxylipins are active lipid mediators and exert specific cellular effects through binding to receptors. Consequently, these mediators play an important role in regulating various biological processes such as inflammation, immunity, vascular functions, as well as a diverse set of homeostatic processes.

Stimulation of cPLA2 and release of ARA has been regarded as a major source of inflammation in cells. ARA interacts with oxidative enzymes such as cyclooxygenase (COX), lipoxygenase (LOX) and cyctochrome P450 (CYP), resulting in synthesis of prostaglandins, leukotrienes and thromboxanes [123,124]. With the exception on lipoxin which is regarded as a protective mediator, most ARA-derived oxylipins exert inflammatory responses through interacting with specific cell receptors. In contrary, DHA interacts with different forms of LOX to form mediators such as resolvins, maresins and neuroprotection D1 (NPD1) [125]. As indicated in brain under ischemic insult, NPD1 exerts protective response and suppresses oxidative insults. ARA accounts for close to half of oxylipins in the brain while less than 20% of oxylipins are attributed to DHA [126]. Interestingly, the brain oxylipin profiles are not altered by changes in dietary n-3 and n-6 PUFA [127]. One possible reason is that these oxylipins are chemically unstable and thus are not stored in tissues but are synthesized de novo only on demand [128]. Nevertheless, pro-inflammatory oxylipins derived from n-6 PUFA are associated with diseases including diabetes [129], kidney disease [130], rheumatoid arthritis and atherosclerosis [131], whereas the n–3 PUFA oxylipins are associated with suppressing inflammation and contributing to the resolution of immune responses [132,133].

Several studies have demonstrated the diverse health benefits of long n-3 PUFAs, such as the role of eicosapentaenoic acid (EPA, 20:5 n-3) and DHA in triglyceride-lowering, anti-inflammatory and antiarrhythmic effects [134,135]. However, when tested in the in vitro conditions, both DHA and ARA increase membrane fluidity, thus, leading to an increase in neurotropic and neuroprotective α-secretase-cleaved soluble APP (sAPPα) in neuronal cells [136]. Dietary n-3 PUFA such as fish oil supplementation have also been shown to increase in EPA and DHA as well as decrease in ARA in erythrocyte membranes [137,138]. Mouse pups nursed by mothers taking DHA supplement showed a large increase in 22:6 (n-3) and decrease in 20:4 (n-6) as compared to controls [139]. Adult mice given a DHA dietary regimen (1% DHA) also showed increase in (n-3) and decrease in (n-6) phospholipid species [140]. In a study with Wistar rats, oral administration of fish oil (38% of DHA and 46% of EPA) for 30 days also showed significant increases in PC and PE with DHA and decreases in PC and PE with ARA in the cortex, hippocampus and striatum [141]. These results indicate that DHA supplement not only increases n-3 fatty acids but suppresses n-6 fatty acids in the membrane phospholipids. In relation with the changes in fatty acids, dietary n-3 PUFA also increases n-3 PUFA derived oxylipins and decreases n-6 PUFA oxylipins in the hippocampus [142]. More studies on analysis of the oxylipins are needed to verify changes in oxylipin patterns upon dietary DHA supplement.

## 12. Peroxidation of ARA and DHA

In addition to reactions with oxygenases, PUFAs are also substrates of non-enzymatic reactions by oxygen free radicals, producing two oxygenated products, namely, 4-hydroxyhexenal (4-HHE) from DHA and 4-hydroxynonenal (4-HNE) from ARA [122]. These bioactive aldehydes are likely present in low levels, but they are metabolic active and able to alter cell metabolism by forming adducts with proteins, phospholipids and nucleic acids [143]. Because different PLA2s show preferences for release of DHA and ARA, it is of interest to follow their peroxidation products. In a study with BV-2 microglia cells, LPS was shown to activate cPLA2 and, thus, releasing ARA and production of 4-HNE [144]. In contrary, increase in 4-HHE but not 4-HNE was observed upon treating cells with DHA. In fact, studies with animal models also demonstrated increase in 4-HHE in brain and other organs upon supplementation with DHA [139]. Interestingly, while dietary DHA alters PUFA in phospholipids in all brain regions, the increase in 4-HHE is found mainly in cerebral cortex and hippocampus, suggesting that these two brain regions are more sensitive in the oxidative pathway [139]. The importance of the hippocampus in lipid peroxidation is demonstrated in a study in which aged mice were supplemented with n-3 PUFA [145]. Changes in lipid peroxidation in the aged mice were associated with enhancement of cellular plasticity in the hippocampus and better object recognition memory. In another study, a short-term n-3 PUFA diet to aged mice showed protection against neuroinflammation and restored spatial memory, events also attributed to special function of the n-3 PUFA in the hippocampal region [146]. In a study with healthy human subjects, supplementation of different levels of DHA also resulted in a dose-related increase in 4-HHE levels in the plasma [147].

Although more studies are needed to investigate the physiological role of 4-HHE in brain, studies with microglial cells indicated that 4-HHE is ten times more potent than DHA in its ability to mitigate LPS-induced inflammation in these cells [144]. In addition, 4-HHE was also 10 times more sensitive to stimulate the antioxidant stress response involving Nrf2 and induction of HO-1, a powerful antioxidant enzyme [144]. Another study with vascular endothelial cells also indicated ability for 4-HHE to enhance the adaptive response pathway involving Nrf2 [148,149]. Although 4-HNE is produced upon stimulation of cPLA2 and peroxidation of ARA in animals and cell models of oxidative stress, the physiological role of this aldehyde in vivo remains to be examined [150,151]. In our studies with cell models, exogenous application of 4-HNE up to 10 µM range showed protective effects similar to the 4-HHE [144]. Using the microglia model, we also reported that 4-HHE was more effective than DHA to counteract activation of cPLA2 and upregulation of iNOS and TNFα by the toxic oligomeric Aβ [52].

The neuroprotective effects of DHA can be, in part, attributed to its bioactive metabolites. The review by Kuda stated over 70 biologically active DHA-derived metabolites derived from enzymic metabolism, oxygenation and conjugation of DHA [152], yet many of their potential effects have not been tested. Due to the recent advancements in organic synthesis [153], it is possible to study the direct effects of some fatty acid-derived metabolites in the biological systems. For example, neuroprotectin D1, an enzymatically oxidized product of DHA, is known to induce neuronal survival and downregulation of amyloidogenic processing in AD cellular models [154]. The 4(RS)-4-F_4t_-neuroprostane (4-F_4t_-NeuroP), a non-enzymatic product derived from oxidation of DHA, has been reported to offer protective effects through decreasing cytochrome c release and caspase 3 activity in ventricular tissue after ischemia/reperfusion [155]. In our recent studies, this compound suppresses oxidative stress, inflammation and mitochondrial dysfunction in LPS-stimulated microglia (unpublished data).

## 13. Phospholipase Products through Lipidomics and Advanced Mass Spectrometry Analysis

### 13.1. Phospholipid Species

The membrane lipid bilayer is comprised of different types of phospholipids, namely PC, PE, PI and PS. In the brain, the PE phospholipids tend to have high levels of alkenylacyl group—plasmalogen. Although plasmalogens are enriched in myelin and the white matter, little is known about their interaction with PLA2 due partly to difficulty to separate the alkenylacyl group from the diacyl group. In the past, phospholipids were separated by one or two dimension thin-layer chromatography [156]. Although most thin-layer protocols lack sensitivity and cannot separate the alkyl/alkenyl linkages from the acyl linkages, improved lipidomic protocols can offer sensitivity and separation of molecular species of individual phospholipid [157]. Using the shotgun lipidomic analysis, our recent study indicated DHA supplements can alter phospholipid species in brain, heart and plasma [140]. Lipidomic analyses of phospholipid species in human plasma have proved to be useful in revealing changes in lipids associated with different neurological diseases and injury [158].

### 13.2. Oxylipins

Improved methods for analysis of oxylipins have unveiled the complexity of these compounds in different cell systems. In the past, radioimmunoassay [159] and enzyme-linked immunosorbent assay [160] are the most widely used techniques for analysis of oxylipins. This approach is problematic because it requires specific antibodies and due to the structural similarity of oxylipins, it is unlikely that antibodies alone can sufficiently distinguish them [161]. Over the years, new chromatography-mass spectrometry-based methods such as liquid chromatography-mass spectrometry (LC–MS) or gas chromatography-mass spectrometry (GC-MS) have developed [162]. LC–MS/MS method offers a cost effective and more sensitive alternative method for oxylipins analysis amidst challenges such as the small mass range occupied by the diverse lipid classes, the structural isomers and their low abundance together with inherent instability in biological fluids [163,164]. Ultrahigh performance liquid chromatography (UHPLC) coupled to tandem MS/MS instruments have demonstrated high resolution, speed and sensitivity for analyzing oxylipins in biological samples and also with good selectivity and low detection limits [162]. Nevertheless, the scope of this approach is limited due to availability of commercial standards. Although recent targeted metabolomic LC–MS approaches with high sensitivity has been used to quantify over 100 oxylipins [164], the combination of UHPLC chromatographic separation and (multiple reaction monitoring) MRM transitions performed on a triple quadrupole (QqQ) mass spectrometer allow 184 eicosanoid metabolites to be separated and quantified in a 5 min running time [165]. In a recent study, Watrous et al. use a non-targeted mass spectrometry approach in conjunction with chemical networking of spectral fragmentation patterns to identify over 500 discrete chemical signals highly consistent with known and putative eicosanoids and related oxylipins in human plasma, including 46 putative molecules not previously described [166]. Future studies with improved technology will advance identification of these oxylipins and aid discovery of their physiological functions.

## 14. Summary and Future Directions

This review has provided a comprehensive coverage of recent knowledge of the three major types of PLA2, namely, cPLA2, iPLA2 and sPLA2. This review also places emphasis on the Yin-Yang metabolism for metabolism of ARA and DHA, leading to production of peroxidative products and oxylipins which can be inflammatory and protective. A summary of the PLA2 subtypes and their properties as well as specific implication on neurological diseases is listed in Table 1. In addition, Table 1 also includes recent studies unveiling the role of lysophospholipids and possible role to enrich brain phospholipids with DHA. Lastly, with the aid of advance proteomics, lipidomics and sensitive LC–MS techniques, future studies will be able to provide new and important information for specific PLA2 and their metabolites in specific cell types and implication for health and diseases.

## Figures and Tables

**Table 1:** Characteristic features of PLA2 and their metabolites.

Title		Activities and Functions	References
A. cPLA2	1.	Phosphorylation by mitogen-activated protein kinases (MAPK)	[8,13,14,15]
	2.	Translocation from cytoplasm to different intracellular components	[9]
	3.	Neuronal excitation and glial activation	[13,14,15,16]
	4.	Preference for release of ARA and inflammatory pathways	[17]
	5.	Involvement in stroke, spinal cord and traumatic brain injury	[27,28,31,32]
	6.	Involvement in AD	[33,34,35,36,37,38,39,40,41,42]
	7.	Activation by oligomeric Aβ	[46,48,49,50,51,52,53]
	8.	Inhibitors—arachidonyl trifluoromethyl ketone (AACOCF3 or ATK)	[17,23,24,53]
B. iPLA2			
	1.	Multiple strings of ankyrin motif—binding with other proteins	[2,56,57]
	2.	iPLA2β—most prominent phospholipase playing a house-keeping role-regulation of mitochondrial function	[58,60,61]
	3.	iPLA2γ—has motif for mitochondrial localization	[67]
	4.	Preference for the release of DHA	[63,64,65]
	5.	Action on cardiolipins	[68,69]
	6.	Regulation of AMPA neurons	[72,73]
	7.	Specific inhibitor, bromoenol lactone (BEL)	[62]
	8.	Mutations of GVI PLA2 gene—infantile neuroaxonal dystrophy (INAD) and neurodegeneration with brain iron accumulation (NBIA)	[57,60,63,74]
C. sPLA2			
	1.	Small molecular weight proteins (20–40 kDa)—more than 10 isoforms	[77]
	2.	Transcriptionally induced upon challenge by toxins and pro-inflammatory cytokines -inflammation	[76,80,83]
	3.	Require high levels of calcium for activity in extracellular milieu	[78]
	4.	Presence in extracellular vesicles secreted from astrocytes	[84,89,90]
	5.	Many mouse strains lack the sPLA2-IIA gene	[85]
	6.	Diseases—AD, cerebral ischemia, spinal cord injury	[93,94,95,96,97,98,99]
	7.	Inhibitors: thioetheramide-phosphorylcholine	[100]
D. Lysophospholipids			
	1.	DHA-LPC—effectively transported to brain	[101,102]
	2.	DHA-lysophospholipids—transported to brain through a specific Na-dependent transporter “Mfsd2A”	[103,104,105]
	3.	Different isoforms of lysophospholipid acyltransferases engage in the “diacylation-reacylation” cycle	[108,109,110]
	4.	LPA—pathways for synthesis	[114,115,116,117]
	5.	LPA—interaction with G-protein-coupled receptors leading to different downstream signaling pathways	[120]
E. ARE, DHA, oxylipins, 4-HNE and 4-HHE			
	1.	Yin-Yang mechanism for the release of ARE and DHA	[122]
	2.	Enzymatic and non-enzymatic free-radical oxidation reactions for production of oxylipins.	
	3.	ARA—interaction with cyclooxygenase (COX), lipoxygenase (LOX) and cyctochrome P450 (CYP) for synthesis of prostaglandins, leukotrienes and thromboxanes, oxylipins that are inflammatory	[123,124]
	4.	DHA—interact with different forms of LOX to form protective mediators such as resolvins, maresins and neuroprotection D1	[125]
	5.	Non-enzymatic free radical reactions—4-hydroxyhexenal (4-HHE) from DHA and 4-hydroxynonenal (4-HNE) from ARA	[122]
	6.	Neuroprostanes	[155]
	7.	Alkenyl aldehydes—bioactive and form adducts with proteins, phospholipids and nucleic acids	[143]
	8.	4-HNE—downstream from stimulation of cPLA2 and ARA	[144]
	9.	4-HHE—increase in brain upon dietary DHA	[139,144]

## References

[1] Kita Y., Shindou H., Shimizu T. (2018). Cytosolic phospholipase A2 and lysophospholipid acyltransferases. Biochim. Biophys. Acta (BBA)—Mol. Cell Biol. Lipids.

[2] Schaloske R.H., Dennis E.A. (2006). The phospholipase A2 superfamily and its group numbering system. Biochim. Biophys. Acta (BBA)—Mol. Cell Biol. Lipids.

[3] Burke J., Dennis E.A. (2009). Phospholipase A2 structure/function, mechanism, and signaling. J. Lipid Res..

[4] Ong W.-Y., Farooqui T., Kokotos G., Farooqui A.A. (2015). Synthetic and Natural Inhibitors of Phospholipases A2: Their Importance for Understanding and Treatment of Neurological Disorders. ACS Chem. Neurosci..

[5] Sun G.Y., Chuang D.Y., Zong Y., Jiang J., Lee J.C.M., Gu Z., Simonyi A. (2014). Role of Cytosolic Phospholipase A2 in Oxidative and Inflammatory Signaling Pathways in Different Cell Types in the Central Nervous System. Mol. Neurobiol..

[6] Sun G.Y., Shelat P.B., Jensen M.B., He Y., Sun A.Y., Simonyi A. (2009). Phospholipases A2 and Inflammatory Responses in the Central Nervous System. NeuroMol. Med..

[7] Dennis E.A., Cao J., Hsu Y.-H., Magrioti V., Kokotos G. (2011). Phospholipase A2 Enzymes: Physical Structure, Biological Function, Disease Implication, Chemical Inhibition, and Therapeutic Intervention. Chem. Rev..

[8] Niknami M., Patel M., Witting P.K., Dong Q. (2009). Molecules in focus: Cytosolic phospholipase A2-α. Int. J. Biochem. Cell Biol..

[9] Sarkar C., Jones J.W., Hegdekar N., Thayer J.A., Kumar A., Faden A.I., Kane M.A., Lipinski M.M. (2019). PLA2G4A/cPLA2-mediated lysosomal membrane damage leads to inhibition of autophagy and neurodegeneration after brain trauma. Autophagy.

[10] Nakamura H., Moriyama Y., Makiyama T., Emori S., Yamashita H., Yamazaki R., Murayama T. (2013). Lactosylceramide Interacts with and Activates Cytosolic Phospholipase A2α. J. Biol. Chem..

[11] Chao C.-C., Gutiérrez-Vázquez C., Rothhammer V., Mayo L., Wheeler M.A., Tjon E.C., Zandee S., Blain M., de Lima K.A., Takenaka M.C. (2019). Metabolic Control of Astrocyte Pathogenic Activity via cPLA2-MAVS. Cell.

[12] Chatterjee S., Balram A., Li W. (2021). Convergence: Lactosylceramide-Centric Signaling Pathways Induce Inflammation, Oxidative Stress, and Other Phenotypic Outcomes. Int. J. Mol. Sci..

[13] Shelat P.B., Chalimoniuk M., Wang J.-H., Strosznajder J.B., Lee J.C., Sun A.Y., Simonyi A., Sun G.Y. (2008). Amyloid beta peptide and NMDA induce ROS from NADPH oxidase and AA release from cytosolic phospholipase A2in cortical neurons. J. Neurochem..

[14] Malada-Edelstein Y.F., Hadad N., Levy R. (2017). Regulatory role of cytosolic phospholipase A2 alpha in the induction of CD40 in microglia. J. Neuroinflamm..

[15] Chuang D.Y., Cui J., Simonyi A., Engel V.A., Chen S., Fritsche K.L., Thomas A.L., Applequist W.L., Folk W.R., Lubahn D.B. (2014). Dietary Sutherlandia and Elderberry Mitigate Cerebral Ischemia-Induced Neuronal Damage and Attenuate p47phox and Phospho-ERK1/2 Expression in Microglial Cells. ASN Neuro.

[16] Yang B., Li R., Liu P.N., Geng X., Mooney B.P., Chen C., Cheng J., Fritsche K.L., Beversdorf D.Q., Lee J.C. (2020). Quantitative Proteomics Reveals Docosahexaenoic Acid-Mediated Neuroprotective Effects in Lipopolysaccharide-Stimulated Microglial Cells. J. Proteome Res..

[17] Riendeau D., Guay J., Weech P., Laliberté F., Yergey J., Li C., Desmarais S., Perrier H., Liu S., Nicoll-Griffith D. (1994). Arachidonyl trifluoromethyl ketone, a potent inhibitor of 85-kDa phospholipase A2, blocks production of arachidonate and 12-hydroxyeicosatetraenoic acid by calcium ionophore-challenged platelets. J. Biol. Chem..

[18] Farooqui A.A., Ong W.Y., Horrocks L.A. (2006). Inhibitors of brain phospholipase A2 activity: Their neuropharmacological effects and thera-peutic importance for the treatment of neurologic disorders. Pharmacol. Rev..

[19] Szaingurten-Solodkin I., Hadad N., Levy R. (2009). Regulatory role of cytosolic phospholipase A2alpha in NADPH oxidase activity and in in-ducible nitric oxide synthase induction by aggregated Abeta1-42 in microglia. Glia.

[20] Anwar K., Voloshyna I., Littlefield M.J., Carsons S.E., Wirkowski P.A., Jaber N.L., Sohn A., Eapen S., Reiss A.B. (2010). COX-2 Inhibition and Inhibition of Cytosolic Phospholipase A2 Increase CD36 Expression and Foam Cell Formation in THP-1 Cells. Lipids.

[21] Meyer A.M., Dwyer-Nield L.D., Hurteau G.J., Keith R.L., O’Leary E., You M., Bonventre J.V., Nemenoff R.A., Malkinson A.M. (2004). Decreased lung tumorigenesis in mice genetically deficient in cytosolic phospholipase A2. Carcinogenesis.

[22] Ishihara K., Miyazaki A., Nabe T., Fushimi H., Iriyama N., Kanai S., Sato T., Uozumi N., Shimizu T., Akiba S. (2012). Group IVA phospholipase A 2 participates in the progression of hepatic fibrosis. FASEB J..

[23] Xiang Y., Wei X., Du P., Zhao H., Liu A., Chen Y. (2019). beta-Arrestin-2-ERK1/2 cPLA2alpha axis mediates TLR4 signaling to influence eicosanoid induction in ischemic brain. FASEB J..

[24] Street I.P., Lin H.K., Laliberte F., Ghomashchi F., Wang Z., Perrier H., Tremblay N.M., Huang Z., Weech P.K., Gelb M.H. (1993). Slow- and tight-binding inhibitors of the 85-kDa human phospholipase A2. Biochemistry.

[25] Schanstra J.P., Luong T.T., Makridakis M., Van Linthout S., Lygirou V., Latosinska A., Alesutan I., Boehme B., Schelski N., Von Lewinski D. (2019). Systems biology identifies cytosolic PLA2 as a target in vascular calcification treatment. JCI Insight.

[26] Kumar S., Sharma S., Kaushik G., Avti P.K., Pandey S., Sarma P., Medhi B., Khanduja K.L. (2018). Therapeutic potential of arachidonyl trifluromethyl ketone, a cytosolic phospholipaseA2 IVA specific inhibitor, in cigarette smoke condensate-induced pathological conditions in alveolar type I & II epithelial cells. Toxicol. In Vitro.

[27] Rodriguez de Turco E.B., Belayev L., Liu Y., Busto R., Parkins N., Bazan N.G., Ginsberg M.D. (2002). Systemic fatty acid responses to transient focal cerebral ischemia: Influence of neuroprotectant therapy with human albumin. J. Neurochem..

[28] Sun G.Y., MacQuarrie R.A. (1989). Deacylation-Reacylation of Arachidonoyl Groups in Cerebral Phospholipids. Ann. N. Y. Acad. Sci..

[29] Golovko S.A., Golovko M. (2018). Plasma Unesterified Fatty-Acid Profile Is Dramatically and Acutely Changed under Ischemic Stroke in the Mouse Model. Lipids.

[30] Shimizu H., Ito A., Sakurada K., Nakamura J., Tanaka K., Komatsu M., Takeda M., Saito K., Endo Y., Kozaki T. (2019). AK106-001616, a Potent and Selective Inhibitor of Cytosolic Phospholipase A2: In Vivo Efficacy for Inflammation, Neuropathic Pain, and Pulmonary Fibrosis. J. Pharmacol. Exp. Ther..

[31] Liu N.-K., Zhang Y.P., Titsworth W.L., Jiang X., Han S., Lu P.-H., Shields C.B., Xu X.-M. (2006). A novel role of phospholipase A2 in mediating spinal cord secondary injury. Ann. Neurol..

[32] Liu N., Deng L., Zhang Y.P., Lu Q., Wang X., Hu J., Oakes E., Bonventre J.V., Shields C.B., Xu X. (2014). Cytosolic phospholipase A2 protein as a novel therapeutic target for spinal cord injury. Ann. Neurol..

[33] Sanchez-Mejia R.O., Mucke L. (2010). Phospholipase A2 and arachidonic acid in Alzheimer’s disease. Biochim. Biophys. Acta (BBA)—Mol. Cell Biol. Lipids.

[34] Lee J.C.-M., Simonyi A., Sun A.Y., Sun G.Y. (2011). Phospholipases A2 and neural membrane dynamics: Implications for Alzheimer’s disease. J. Neurochem..

[35] Sun G.Y., He Y., Chuang D.Y., Lee J.C., Gu Z., Simonyi A., Sun A.Y. (2012). Integrating Cytosolic Phospholipase A2 with Oxidative/Nitrosative Signaling Pathways in Neurons: A Novel Therapeutic Strategy for AD. Mol. Neurobiol..

[36] Schaeffer E.L., Forlenza O.V., Gattaz W.F. (2009). Phospholipase A2 activation as a therapeutic approach for cognitive enhancement in ear-ly-stage Alzheimer disease. Psychopharmacology.

[37] Sanchez-Mejia R.O., Newman J., Toh S., Yu G.-Q., Zhou Y., Halabisky B., Cissé M., Scearce-Levie K., Cheng I.H., Gan L. (2008). Phospholipase A2 reduction ameliorates cognitive deficits in a mouse model of Alzheimer’s disease. Nat. Neurosci..

[38] Feng C., Bao X., Shan L., Ling Y., Ding Y., Wang J., Cao Y., Wang Q., Cui W., Xu S. (2020). Calcium-Sensing Receptor Mediates beta-Amyloid-Induced Synaptic Formation Impairment and Cognitive Deficits via Reg-ulation of Cytosolic Phospholipase A2/Prostaglandin E2 Metabolic Pathway. Front. Aging Neurosci..

[39] Kaya I., Jennische E., Lange S., Tarik Baykal A., Malmberg P., Fletcher J.S. (2020). Brain region-specific amyloid plaque-associated myelin lipid loss, APOE deposition and disruption of the myelin sheath in familial Alzheimer’s disease mice. J. Neurochem..

[40] Mehla J., Lacoursiere S.G., Lapointe V., McNaughton B.L., Sutherland R.J., McDonald R.J., Mohajerani M.H. (2018). Age-dependent behavioral and biochemical characterization of single APP knock-in mouse (APPNL-G-F/NL-G-F) model of Alzheimer’s disease. Neurobiol. Aging.

[41] Sakakibara Y., Sekiya M., Saito T., Saido T.C., Iijima K.M. (2019). Amyloid-beta plaque formation and reactive gliosis are required for induction of cognitive deficits in App knock-in mouse models of Alzheimer’s disease. BMC Neurosci..

[42] Emre C., Do K.V., Jun B., Hjorth E., Alcalde S.G., Kautzmann M.-A.I., Gordon W.C., Nilsson P., Bazan N.G., Schultzberg M. (2021). Age-related changes in brain phospholipids and bioactive lipids in the APP knock-in mouse model of Alzheimer’s disease. Acta Neuropathol. Commun..

[43] Granger M.W., Liu H., Fowler C., Blanchard A.P., Taylor M.W., Sherman S.P.M., Xu H., Le W., Bennett S.A.L. (2018). Distinct disruptions in Land’s cycle remodeling of glycerophosphocholines in murine cortex mark symptomatic onset and progression in two Alzheimer’s disease mouse models. J. Neurochem..

[44] Pérez-González M., Mendioroz M., Badesso S., Sucunza D., Roldan M., Espelosín M., Ursua S., Luján R., Cuadrado-Tejedor M., Garcia-Osta A. (2020). PLA2G4E, a candidate gene for resilience in Alzheimer’s disease and a new target for dementia treatment. Prog. Neurobiol..

[45] Dahlgren K.N., Manelli A.M., Stine W.B., Baker L.K., Krafft G.A., LaDu M.J. (2002). Oligomeric and fibrillar species of amyloid-beta peptides differentially affect neuronal viability. J. Biol. Chem..

[46] Hicks J.B., Lai Y., Sheng W., Yang X., Zhu D., Sun G.Y., Lee J.C.-M. (2008). Amyloid-β peptide induces temporal membrane biphasic changes in astrocytes through cytosolic phospholipase A2. Biochim. Biophys. Acta (BBA)—Biomembr..

[47] Zhu D., Lai Y., Shelat P.B., Hu C., Sun G.Y., Lee J.C. (2006). Phospholipases A2 mediate amyloid-beta peptide-induced mitochondrial dysfunction. J. Neurosci..

[48] Sagy-Bross C., Kasianov K., Solomonov Y., Braiman A., Friedman A., Hadad N., Lévy R. (2015). The role of cytosolic phospholipase A2α in amyloid precursor protein induction by amyloid beta1-42: Implication for neurodegeneration. J. Neurochem..

[49] Sagy-Bross C., Hadad N., Levy R. (2013). Cytosolic phospholipase A2alpha upregulation mediates apoptotic neuronal death induced by ag-gregated amyloid-beta peptide1-42. Neurochem. Int..

[50] Desbene C., Malaplate-Armand C., Youssef I., Garcia P., Stenger C., Sauvee M., Fischer N., Rimet D., Koziel V., Escanye M.C. (2012). Critical role of cPLA2 in Abeta oligomer-induced neurodegeneration and memory deficit. Neurobiol. Aging.

[51] Teng T., Dong L., Ridgley D.M., Ghura S., Tobin M.K., Sun G.Y., LaDu M.J., Lee J.C. (2019). Cytosolic Phospholipase A2 Facilitates Oligomeric Amyloid-beta Peptide Association with Microglia via Regulation of Mem-brane-Cytoskeleton Connectivity. Mol. Neurobiol..

[52] Geng X., Yang B., Li R., Teng T., Ladu M.J., Sun G.Y., Greenlief C.M., Lee J.C. (2020). Effects of Docosahexaenoic Acid and Its Peroxidation Product on Amyloid-beta Peptide-Stimulated Microglia. Mol. Neurobiol..

[53] Teng T., Ridgley D.M., Tsoy A., Sun G.Y., Askarova S., Lee J.C. (2018). Azelnidipine Attenuates the Oxidative and NFκB Pathways in Amyloid-β-Stimulated Cerebral Endothelial Cells. ACS Chem. Neurosci..

[54] Butterfield D.A., Mattson M.P. (2020). Apolipoprotein E and oxidative stress in brain with relevance to Alzheimer’s disease. Neurobiol. Dis..

[55] Wang S., Li B., Solomon V., Fonteh A., Rapoport S.I., Bennett D.A., Arvanitakis Z., Chui H.C., Miller C., Sullivan P.M. (2021). Calcium-dependent cytosolic phospholipase A2 activation is implicated in neuroinflammation and oxidative stress associated with ApoE4. Mol. Neurodegener..

[56] Turk J., Song H., Wohltmann M., Frankfater C., Lei X., Ramanadham S. (2020). Metabolic Effects of Selective Deletion of Group VIA Phospholipase A2 from Macrophages or Pancreatic Islet Beta-Cells. Biomolecules.

[57] Turk J., White T.D., Nelson A.J., Lei X., Ramanadham S. (2019). iPLA2beta and its role in male fertility, neurological disorders, metabolic disorders, and inflammation. Biochim. Biophys. Acta—Mol. Cell Biol. Lipids.

[58] Balboa M.A., Varela-Nieto I., Lucas K.K., Dennis E.A. (2002). Expression and function of phospholipase A2 in brain. FEBS Lett..

[59] Mendes C.T., Gattaz W.F., Schaeffer E.L., Forlenza O.V. (2005). Modulation of phospholipase A2 activity in primary cultures of rat cortical neurons. J. Neural Transm..

[60] Kinghorn K.J., Castillo-Quan J.I., Bartolome F., Angelova P.R., Li L., Pope S., Cocheme H.M., Khan S., Asghari S., Bhatia K.P. (2015). Loss ofPLA2G6leads to elevated mitochondrial lipid peroxidation and mitochondrial dysfunction. Brain.

[61] Strokin M., Reiser G. (2016). Mitochondria from a mouse model of the human infantile neuroaxonal dystrophy (INAD) with genetic defects in VIA iPLA2 have disturbed Ca^2+^ regulation with reduction in Ca^2+^ capacity. Neurochem. Int..

[62] St-Gelais F., Menard C., Congar P., Trudeau L.E., Massicotte G. (2004). Postsynaptic injection of calcium-independent phospholipase A2 inhibitors selectively increases AMPA receptor-mediated synaptic transmission. Hippocampus.

[63] Blanchard H., Taha A.Y., Cheon Y., Kim H.-W., Turk J., Rapoport S.I. (2014). iPLA2β Knockout Mouse, a Genetic Model for Progressive Human Motor Disorders, Develops Age-Related Neuropathology. Neurochem. Res..

[64] Basselin M., Rosa A.O., Ramadan E., Cheon Y., Chang L., Chen M., Greenstein D., Wohltmann M., Turk J., Rapoport S.I. (2010). Imaging decreased brain docosahexaenoic acid metabolism and signaling in iPLA(2)beta (VIA)-deficient mice. J. Lipid Res..

[65] Strokin M., Sergeeva M., Reiser G. (2003). Docosahexaenoic acid and arachidonic acid release in rat brain astrocytes is mediated by two separate isoforms of phospholipase A2and is differently regulated by cyclic AMP and Ca^2+^. Br. J. Pharmacol..

[66] Cheon Y., Kim H.W., Igarashi M., Modi H.R., Chang L., Ma K., Greenstein D., Wohltmann M., Turk J., Rapoport S.I. (2012). Disturbed brain phospholipid and docosahexaenoic acid metabolism in calcium-independent phospholipase A(2)-VIA (iPLA(2)beta)-knockout mice. Biochim. Biophys. Acta.

[67] Hara S., Yoda E., Sasaki Y., Nakatani Y., Kuwata H. (2019). Calcium-independent phospholipase A2gamma (iPLA2gamma) and its roles in cellular functions and diseases. Biochim. Biophys. Acta—Mol. Cell Biol. Lipids.

[68] Jaburek M., Pruchova P., Holendova B., Galkin A., Jezek P. (2021). Antioxidant Synergy of Mitochondrial Phospholipase PNPLA8/iPLA2gamma with Fatty Acid-Conducting SLC25 Gene Family Transporters. Antioxidants.

[69] Liu G.Y., Moon S.H., Jenkins C.M., Li M., Sims H.F., Guan S., Gross R.W. (2017). The phospholipase iPLA2gamma is a major mediator releasing oxidized aliphatic chains from cardiolipin, integrating mito-chondrial bioenergetics and signaling. J. Biol. Chem..

[70] Mancuso D.J., Jenkins C.M., Gross R.W. (2000). The genomic organization, complete mRNA sequence, cloning, and expression of a novel human intracellular membrane-associated calcium-independent phospholipase A(2). J. Biol. Chem..

[71] Takata-Tanaka H., Takeya R., Sumimoto H. (2000). A Novel Intracellular Membrane-Bound Calcium-Independent Phospholipase A2. Biochem. Biophys. Res. Commun..

[72] Allyson J., Bi X., Baudry M., Massicotte G. (2012). Maintenance of Synaptic Stability Requires Calcium-Independent Phospholipase A2Activity. Neural Plast..

[73] Ménard C., Chartier E., Patenaude C., Robinson P., Cyr M., Baudry M., Massicotte G. (2007). Calcium-independent phospholipase A2 influences AMPA-mediated toxicity of hippocampal slices by regulating the GluR1 subunit in synaptic membranes. Hippocampus.

[74] Fusco C., Frattini D., Panteghini C., Pascarella R., Garavaglia B. (2014). A Case of Infantile Neuroaxonal Dystrophy of Neonatal Onset. J. Child Neurol..

[75] Mori A., Hatano T., Inoshita T., Shiba-Fukushima K., Koinuma T., Meng H., Kubo S.-I., Spratt S., Cui C., Yamashita C. (2019). Parkinson’s disease-associated iPLA2-VIA/PLA2G6 regulates neuronal functions and α-synuclein stability through membrane remodeling. Proc. Natl. Acad. Sci. USA.

[76] Friend S.F., Nachnani R., Powell S.B., Risbrough V.B. (2020). C-Reactive Protein: Marker of risk for post-traumatic stress disorder and its potential for a mechanistic role in trauma response and recovery. Eur. J. Neurosci..

[77] Khan M.I., Hariprasad G. (2020). Human Secretary Phospholipase A2 Mutations and Their Clinical Implications. J. Inflamm. Res..

[78] Talib L.L., Diniz B., Zainaghi I.A., Forlenza O.V., Gattaz W.F. (2012). A radioenzymatic assay to identify three groups of phospholipase A2 in platelets. Prostaglandins Leukot. Essent. Fat. Acids.

[79] Duchez A.-C., Boudreau L.H., Naika G.S., Rousseau M., Cloutier N., Levesque T., Gelb M.H., Boilard E. (2019). Respective contribution of cytosolic phospholipase A2α and secreted phospholipase A2 IIA to inflammation and eicosanoid production in arthritis. Prostaglandins Other Lipid Mediat..

[80] Dore E., Boilard E. (2018). Roles of secreted phospholipase A2 group IIA in inflammation and host defense. Biochim. Biophys. Acta (BBA)—Mol. Cell Biol. Lipids.

[81] Murakami M., Yamamoto K., Taketomi Y. (2018). Phospholipase A2 in skin biology: New insights from gene-manipulated mice and lipidomics. Inflamm. Regen..

[82] Murakami M., Miki Y., Sato H., Murase R., Taketomi Y., Yamamoto K. (2019). Group IID, IIE, IIF and III secreted phospholipase A2s. Biochim. Biophys. Acta—Mol. Cell Biol. Lipids.

[83] Sheng W., Zong Y., Mohammad A., Ajit D., Cui J., Han D., Hamilton J.L., Simonyi A., Sun A.Y., Gu Z. (2011). Proinflammatory cytokines and lipopolysaccharide induce changes in cell morphology, and upregulation of ERK1/2, iNOS and sPLA2-IIA expression in astrocytes and microglia. J. Neuroinflamm..

[84] Villanueva E., Little J.P., Lambeau G., Klegeris A. (2012). Secreted phospholipase A2 group IIA is a neurotoxin released by stimulated human glial cells. Mol. Cell. Neurosci..

[85] Kennedy B.P., Payette P., Mudgett J., Vadas P., Pruzanski W., Kwan M., Tang C., Rancourt D.E., Cromlish W.A. (1995). A Natural Disruption of the Secretory Group II Phospholipase A2 Gene in Inbred Mouse Strains. J. Biol. Chem..

[86] Grass D.S., Felkner R.H., Chiang M.Y., Wallace R.E., Nevalainen T.J., Bennett C.F., Swanson M.E. (1996). Expression of human group II PLA2 in transgenic mice results in epidermal hyperplasia in the absence of inflammatory infiltrate. J. Clin. Investig..

[87] Ivandic B., Castellani L.W., Wang X.P., Qiao J.H., Mehrabian M., Navab M., Fogelman A.M., Grass D.S., Swanson M.E., de Beer M.C. (1999). Role of group II secretory phospholipase A2 in atherosclerosis: 1. Increased atherogenesis and altered lipoproteins in transgenic mice expressing group IIa phospholipase A2. Arterioscler. Thromb. Vasc. Biol..

[88] Bonventre J.V. (2004). Cytosolic phospholipase A2α reigns supreme in arthritis and bone resorption. Trends Immunol..

[89] Subra C., Grand D., Laulagnier K., Stella A., Lambeau G., Paillasse M., De Medina P., Monsarrat B., Perret B., SilventePoirot S. (2010). Exosomes account for vesicle-mediated transcellular transport of activatable phospholipases and prostaglandins. J. Lipid Res..

[90] Akinkuolie A.O., Lawler P.R., Chu A.Y., Caulfield M., Mu J., Ding B., Nyberg F., Glynn R.J., Ridker P.M., Hurt-Camejo E. (2019). Group IIA Secretory Phospholipase A2, Vascular Inflammation, and Incident Cardiovascular Disease. Arterioscler. Thromb. Vasc. Biol..

[91] Papadopoulos S., Kazepidou E., Antonelou M.H., Leondaritis G., Tsapinou A., Koulouras V.P., Avgeropoulos A., Nakos G., Lekka M.E. (2020). Secretory Phospholipase A2-IIA Protein and mRNA Pools in Extracellular Vesicles of Bronchoalveolar Lavage Fluid from Patients with Early Acute Respiratory Distress Syndrome: A New Perception in the Dissemination of Inflammation?. Pharmaceuticals.

[92] Zhang L., Xia R., Jia J., Wang L., Li K., Li Y., Zhang J. (2018). Oleanolic acid protects against cognitive decline and neuroinflammation-mediated neurotoxicity by blocking secretory phospholipase A2 IIA-activated calcium signals. Mol. Immunol..

[93] Moses G.S., Jensen M.D., Lue L.-F., Walker D.G., Sun A.Y., Simonyi A., Sun G.Y. (2006). Secretory PLA2-IIA: A new inflammatory factor for Alzheimer’s disease. J. Neuroinflamm..

[94] Yang X., Sheng W., Ridgley D.M., Haidekker M.A., Sun G.Y., Lee J.C. (2015). Astrocytes regulate α-secretase-cleaved soluble amyloid precursor protein secretion in neuronal cells: Involvement of group IIA secretory phospholipase A2. Neuroscience.

[95] Lin T.-N., Wang Q., Simonyi A., Chen J.-J., Cheung W.-M., He Y.Y., Xu J., Sun A.Y., Hsu C.Y., Sun G.Y. (2004). Induction of secretory phospholipase A2 in reactive astrocytes in response to transient focal cerebral ischemia in the rat brain. J. Neurochem..

[96] Wang Q., Sun A.Y., Pardeike J., Müller R.H., Simonyi A., Sun G.Y. (2009). Neuroprotective effects of a nanocrystal formulation of sPLA2 inhibitor PX-18 in cerebral ischemia/reperfusion in gerbils. Brain Res..

[97] Titsworth W.L., Cheng X., Ke Y., Deng L., Burckardt K.A., Pendleton C., Liu N.K., Shao H., Cao Q.L., Xu X.M. (2009). Differential expression of sPLA2 following spinal cord injury and a functional role for sPLA2-IIA in mediating oli-godendrocyte death. Glia.

[98] Quindlen-Hotek J.C., Kartha S., Winkelstein B.A. (2020). Immediate inhibition of spinal secretory phospholipase A2 prevents the pain and elevated spinal neuronal hyperexcitability and neuroimmune regulatory genes that develop with nerve root compression. NeuroReport.

[99] Kartha S., Weisshaar C.L., Philips B.H., Winkelstein B.A. (2018). Pre-treatment with Meloxicam Prevents the Spinal Inflammation and Oxidative Stress in DRG Neurons that Accompany Painful Cervical Radiculopathy. Neuroscience.

[100] Kartha S., Yan L., Ita M.E., Amirshaghaghi A., Luo L., Wei Y., Tsourkas A., Winkelstein B.A., Cheng Z. (2020). Phospholipase A2 Inhibitor-Loaded Phospholipid Micelles Abolish Neuropathic Pain. ACS Nano.

[101] Sugasini D., Thomas R., Yalagala P.C., Tai L.M., Subbaiah P.V. (2017). Dietary docosahexaenoic acid (DHA) as lysophosphatidylcholine, but not as free acid, enriches brain DHA and improves memory in adult mice. Sci. Rep..

[102] Sugasini D., Yalagala P.C.R., Goggin A., Tai L.M., Subbaiah P.V. (2019). Enrichment of brain docosahexaenoic acid (DHA) is highly dependent upon the molecular carrier of dietary DHA: Lyso-phosphatidylcholine is more efficient than either phosphatidylcholine or triacylglycerol. J. Nutr. Biochem..

[103] Chan J.P., Wong B.H., Chin C.F., Galam D.L.A., Foo J.C., Wong L.C., Ghosh S., Wenk M.R., Cazenave-Gassiot A., Silver D.L. (2018). The lysolipid transporter Mfsd2a regulates lipogenesis in the developing brain. PLoS Biol..

[104] Nguyen L.N., Ma D., Shui G., Wong P., Cazenave-Gassiot A., Zhang X., Wenk M.R., Goh E., Silver D. (2014). Mfsd2a is a transporter for the essential omega-3 fatty acid docosahexaenoic acid. Nature.

[105] Cater R.J., Chua G.L., Erramilli S.K., Keener J.E., Choy B.C., Tokarz P., Chin C.F., Quek D.Q.Y., Kloss B., Pepe J.G. (2021). Structural basis of omega-3 fatty acid transport across the blood–brain barrier. Nature.

[106] Scala M., Chua G.L., Chin C.F., Alsaif H.S., Borovikov A., Riazuddin S., Riazuddin S., Manzini M.C., Severino M., Kuk A. (2020). Biallelic MFSD2A variants associated with congenital microcephaly, developmental delay, and recognizable neuroimaging features. Eur. J. Hum. Genet..

[107] Harel T., Quek D.Q.Y., Wong B.H., Cazenave-Gassiot A., Wenk M.R., Fan H., Berger I., Shmueli D., Shaag A., Silver D.L. (2018). Homozygous mutation in MFSD2A, encoding a lysolipid transporter for docosahexanoic acid, is associated with microcephaly and hypomyelination. Neurogenetics.

[108] Pérez-Chacón G., Astudillo A.M., Balgoma D., Balboa M.A., Balsinde J. (2009). Control of free arachidonic acid levels by phospholipases A2 and lysophospholipid acyltransferases. Biochim. Biophys. Acta (BBA)—Mol. Cell Biol. Lipids.

[109] Murphy R.C., Folco G. (2019). Lysophospholipid acyltransferases and leukotriene biosynthesis: Intersection of the Lands cycle and the ara-chidonate PI cycle. J. Lipid Res..

[110] Pérez-Chacón G., Astudillo A.M., Ruipérez V., Balboa M.A., Balsinde J. (2009). Signaling Role for Lysophosphatidylcholine Acyltransferase 3 in Receptor-Regulated Arachidonic Acid Reacylation Reactions in Human Monocytes. J. Immunol..

[111] Johansen A., Rosti R.O., Musaev D., Sticca E., Harripaul R., Zaki M., Caglayan A.O., Azam M., Sultan T., Froukh T. (2016). Mutations in MBOAT7, Encoding Lysophosphatidylinositol Acyltransferase I, Lead to Intellectual Disability Accompa-nied by Epilepsy and Autistic Features. Am. J. Hum. Genet..

[112] Sundaram J.R., Chan E.S., Poore C.P., Pareek T., Cheong W.F., Shui G., Tang N., Low C.-M., Wenk M.R., Kesavapany S. (2012). Cdk5/p25-Induced Cytosolic PLA2-Mediated Lysophosphatidylcholine Production Regulates Neuroinflammation and Triggers Neurodegeneration. J. Neurosci..

[113] Yatomi Y., Kurano M., Ikeda H., Igarashi K., Kano K., Aoki J. (2018). Lysophospholipids in laboratory medicine. Proc. Jpn. Acad. Ser. B.

[114] Aoki J., Inoue A., Okudaira S. (2008). Two pathways for lysophosphatidic acid production. Biochim. Biophys. Acta.

[115] Nakasaki T., Tanaka T., Okudaira S., Hirosawa M., Umemoto E., Otani K., Jin S., Bai Z., Hayasaka H., Fukui Y. (2008). Involvement of the Lysophosphatidic Acid-Generating Enzyme Autotaxin in Lymphocyte-Endothelial Cell Interactions. Am. J. Pathol..

[116] Herr D.R., Chew W.S., Satish R.L., Ong W.-Y. (2019). Pleotropic Roles of Autotaxin in the Nervous System Present Opportunities for the Development of Novel Therapeutics for Neurological Diseases. Mol. Neurobiol..

[117] Ma L., Uchida H., Nagai J., Inoue M., Aoki J., Ueda H. (2010). Evidence for De Novo Synthesis of Lysophosphatidic Acid in the Spinal Cord through Phospholipase A2 and Autotaxin in Nerve Injury-Induced Neuropathic Pain. J. Pharmacol. Exp. Ther..

[118] Hao Y., Guo M., Feng Y., Dong Q., Cui M. (2020). Lysophospholipids and Their G-Coupled Protein Signaling in Alzheimer’s Disease: From Physiological Performance to Patho-logical Impairment. Front. Mol. Neurosci..

[119] Blaho V.A., Chun J. (2018). ‘Crystal’ Clear? Lysophospholipid Receptor Structure Insights and Controversies. Trends Pharmacol. Sci..

[120] Ramesh S., Govindarajulu M., Suppiramaniam V., Moore T., Dhanasekaran M. (2018). Autotaxin(−)Lysophosphatidic Acid Signaling in Alzheimer’s Disease. Int. J. Mol. Sci..

[121] Fransson J., Gomez-Conde A.I., Romero-Imbroda J., Fernandez O., Leyva L., de Fonseca F.R., Chun J., Louapre C., Van-Evercooren A.B., Zujovic V. (2021). Activation of Macrophages by Lysophosphatidic Acid through the Lysophosphatidic Acid Receptor 1 as a Novel Mecha-nism in Multiple Sclerosis Pathogenesis. Mol. Neurobiol..

[122] Yang B., Fritsche K.L., Beversdorf D.Q., Gu Z., Lee J.C., Folk W.R., Greenlief C.M., Sun G.Y. (2019). Yin-Yang Mechanisms Regulating Lipid Peroxidation of Docosahexaenoic Acid and Arachidonic Acid in the Central Nervous System. Front. Neurol..

[123] Schebb N.H., Ostermann A.I., Yang J., Hammock B.D., Hahn A., Schuchardt J.P. (2014). Comparison of the effects of long-chain omega-3 fatty acid supplementation on plasma levels of free and esterified oxylipins. Prostaglandins Other Lipid Mediat..

[124] Buczynski M.W., Dumlao D.S., Dennis E.A. (2009). Thematic Review Series: Proteomics. An integrated omics analysis of eicosanoid biology. J. Lipid Res..

[125] Palacios-Pelaez R., Lukiw W.J., Bazan N.G. (2010). Omega-3 Essential Fatty Acids Modulate Initiation and Progression of Neurodegenerative Disease. Mol. Neurobiol..

[126] Ferdouse A., Leng S., Winter T., Aukema H.M. (2019). Dietary n-6 and n-3 PUFA alter the free oxylipin profile differently in male and female rat hearts. Br. J. Nutr..

[127] Ferdouse A., Leng S., Winter T., Aukema H.M. (2019). The Brain Oxylipin Profile Is Resistant to Modulation by Dietary n-6 and n-3 Polyunsaturated Fatty Acids in Male and Female Rats. Lipids.

[128] Tourdot B.E., Ahmed I., Holinstat M. (2014). The emerging role of oxylipins in thrombosis and diabetes. Front. Pharmacol..

[129] Grapov D., Adams S.H., Pedersen T.L., Garvey W.T., Newman J.W. (2012). Type 2 Diabetes Associated Changes in the Plasma Non-Esterified Fatty Acids, Oxylipins and Endocannabinoids. PLoS ONE.

[130] Zivkovic A.M., Yang J., Georgi K., Hegedus C., Nording M., O’Sullivan A., German J.B., Hogg R.J., Weiss R.H., Bay C. (2012). Serum oxylipin profiles in IgA nephropathy patients reflect kidney functional alterations. Metabolomics.

[131] Jira W., Spiteller G., Richter A. (1997). Increased levels of lipid oxidation products in low density lipoproteins of patients suffering from rheumatoid arthritis. Chem. Phys. Lipids.

[132] Duffield J.S., Hong S., Vaidya V.S., Lu Y., Fredman G., Serhan C.N., Bonventre J.V. (2006). Resolvin D Series and Protectin D1 Mitigate Acute Kidney Injury. J. Immunol..

[133] Aoki H., Hisada T., Ishizuka T., Utsugi M., Kawata T., Shimizu Y., Okajima F., Dobashi K., Mori M. (2008). Resolvin E1 dampens airway inflammation and hyperresponsiveness in a murine model of asthma. Biochem. Biophys. Res. Commun..

[134] O’Connell T.D., Mason R.P., Budoff M.J., Navar A.M., Shearer G.C. (2020). Mechanistic insights into cardiovascular protection for omega-3 fatty acids and their bioactive lipid metabolites. Eur. Heart J. Suppl..

[135] Hashimoto M., Hossain S., Al Mamun A., Matsuzaki K., Arai H. (2016). Docosahexaenoic acid: One molecule diverse functions. Crit. Rev. Biotechnol..

[136] Yang X., Sheng W., Sun G.Y., Lee J.C.-M. (2011). Effects of fatty acid unsaturation numbers on membrane fluidity and α-secretase-dependent amyloid precursor protein processing. Neurochem. Int..

[137] Schuchardt J.P., Schmidt S., Kressel G., Willenberg I., Hammock B.D., Hahn A., Schebb N.H. (2014). Modulation of blood oxylipin levels by long-chain omega-3 fatty acid supplementation in hyper- and normolipidemic men. Prostaglandins Leukot. Essent. Fat. Acids.

[138] Neubronner J., Schuchardt J.P., Kressel G., Merkel M., Von Schacky C., Hahn A. (2010). Enhanced increase of omega-3 index in response to long-term n-3 fatty acid supplementation from triacylglycerides versus ethyl esters. Eur. J. Clin. Nutr..

[139] Yang B., Li R., Woo T., Browning J.D., Song H., Gu Z., Cui J., Lee J.C., Fritsche K.L., Beversdorf D.Q. (2019). Maternal Dietary Docosahexaenoic Acid Alters Lipid Peroxidation Products and (n-3)/(n-6) Fatty Acid Balance in Offspring Mice. Metabolites.

[140] Sun G.Y., Appenteng M.K., Li R., Woo T., Yang B., Qin C., Pan M., Cieślik M., Cui J., Fritsche K.L. (2020). Docosahexaenoic Acid (DHA) Supplementation Alters Phospholipid Species and Lipid Peroxidation Products in Adult Mouse Brain, Heart, and Plasma. NeuroMol. Med..

[141] Lamaziere A., Richard D., Barbe U., Kefi K., Bausero P., Wolf C., Visioli F. (2011). Differential distribution of DHA-phospholipids in rat brain after feeding: A lipidomic approach. Prostaglandins Leukot. Essent. Fat. Acids.

[142] Rey C., Delpech J., Madore C., Nadjar A., Greenhalgh A., Amadieu C., Aubert A., Pallet V., Vaysse C., Layé S. (2018). Dietary n-3 long chain PUFA supplementation promotes a pro-resolving oxylipin profile in the brain. Brain Behav. Immun..

[143] Cohen G., Riahi Y., Sunda V., Deplano S., Chatgilialoglu C., Ferreri C., Kaiser N., Sasson S. (2013). Signaling properties of 4-hydroxyalkenals formed by lipid peroxidation in diabetes. Free. Radic. Biol. Med..

[144] Yang B., Li R., Michael Greenlief C., Fritsche K.L., Gu Z., Cui J., Lee J.C., Beversdorf D.Q., Sun G.Y. (2018). Unveiling anti-oxidative and anti-inflammatory effects of docosahexaenoic acid and its lipid peroxidation product on lipo-polysaccharide-stimulated BV-2 microglial cells. J. Neuroinflamm..

[145] Cutuli D., De Bartolo P., Caporali P., Laricchiuta D., Foti F., Ronci M., Rossi C., Neri C., Spalletta G., Caltagirone C. (2014). n-3 polyunsaturated fatty acids supplementation enhances hippocampal functionality in aged mice. Front. Aging Neurosci..

[146] Labrousse V.F., Nadjar A., Joffre C., Costes L., Aubert A., Gregoire S., Bretillon L., Laye S. (2012). Short-term long chain omega3 diet protects from neuroinflammatory processes and memory impairment in aged mice. PLoS ONE.

[147] Calzada C., Colas R., Guillot N., Guichardant M., Laville M., Véricel E., Lagarde M. (2010). Subgram daily supplementation with docosahexaenoic acid protects low-density lipoproteins from oxidation in healthy men. Atherosclerosis.

[148] Ishikado A., Nishio Y., Morino K., Ugi S., Kondo H., Makino T., Kashiwagi A., Maegawa H. (2010). Low concentration of 4-hydroxy hexenal increases heme oxygenase-1 expression through activation of Nrf2 and anti-oxidative activity in vascular endothelial cells. Biochem. Biophys. Res. Commun..

[149] Nakagawa F., Morino K., Ugi S., Ishikado A., Kondo K., Sato D., Konno S., Nemoto K., Kusunoki C., Sekine O. (2014). 4-Hydroxy hexenal derived from dietary n-3 polyunsaturated fatty acids induces anti-oxidative enzyme heme oxy-genase-1 in multiple organs. Biochem. Biophys. Res. Commun..

[150] Li Y., Liu S.-L., Qi S.-H. (2018). ALDH2 Protects Against Ischemic Stroke in Rats by Facilitating 4-HNE Clearance and AQP4 DownRegulation. Neurochem. Res..

[151] Hall E.D., Wang J.A., Miller D.M., Cebak J.E., Hill R.L. (2018). Newer pharmacological approaches for antioxidant neuroprotection in traumatic brain injury. Neuropharmacology.

[152] Kuda O. (2017). Bioactive metabolites of docosahexaenoic acid. Biochimie.

[153] Galano J.-M., Lee Y.Y., Oger C., Vigor C., Vercauteren J., Durand T., Giera M., Lee J.C.-Y. (2017). Isoprostanes, neuroprostanes and phytoprostanes: An overview of 25 years of research in chemistry and biology. Prog. Lipid Res..

[154] Stark D.T., Bazan N.G. (2011). Neuroprotectin D1 Induces Neuronal Survival and Downregulation of Amyloidogenic Processing in Alzheimer’s Disease Cellular Models. Mol. Neurobiol..

[155] Roy J., Fauconnier J., Oger C., Farah C., Angebault-Prouteau C., Thireau J., Bideaux P., Scheuermann V., Bultel-Ponce V., Demion M. (2017). Non-enzymatic oxidized metabolite of DHA, 4(RS)-4-F4t-neuroprostane protects the heart against reperfusion injury. Free Radic. Biol. Med..

[156] Sun G.Y., Lin T.-N. (1989). Time course for labeling of brain membrane phosphoinositides and other phospholipids after intracerebral injection of [32P]-ATP. Evaluation by an improved HPTLC procedure. Life Sci..

[157] Wang M., Han X. (2016). Advanced Shotgun Lipidomics for Characterization of Altered Lipid Patterns in Neurodegenerative Diseases and Brain Injury. Methods Mol. Biol..

[158] Huguenard C.J.C., Cseresznye A., Evans J.E., Oberlin S., Langlois H., Ferguson S., Darcey T., Nkiliza A., Dretsch M., Mullan M. (2020). Plasma Lipidomic Analyses in Cohorts With mTBI and/or PTSD Reveal Lipids Differentially Associated With Diag-nosis and APOE epsilon4 Carrier Status. Front. Physiol..

[159] Dray F., Charbonnel B., Maclouf J. (1975). Radioimmunoassay of prostaglandins Falpha, E1 and E2 in human plasma. Eur. J. Clin. Investig..

[160] Quinn J.V., Bilgrami S., Seidel G.J., Slotman G.J. (1996). Evaluation of enzyme-linked immunosorbent assays for quantitation of eicosanoid mediators of sepsis syndrome. Shock.

[161] Wang D., DuBois R.N. (2007). Measurement of Eicosanoids in Cancer Tissues. Methods Enzymol..

[162] Liakh I., Pakiet A., Sledzinski T., Mika A. (2020). Methods of the Analysis of Oxylipins in Biological Samples. Molecules.

[163] Hinz C., Liggi S., Mocciaro G., Jung S., Induruwa I., Pereira M., Bryant C.E., Meckelmann S.W., O’Donnell V.B., Farndale R.W. (2019). Comprehensive UHPLC Ion Mobility Quadrupole Time-of-Flight Method for Profiling and Quantification of Eicosanoids, Other Oxylipins, and Fatty Acids. Anal. Chem..

[164] Willenberg I., Ostermann A.I., Schebb N.H. (2015). Targeted metabolomics of the arachidonic acid cascade: Current state and challenges of LC–MS analysis of oxylipins. Anal. Bioanal. Chem..

[165] Wang Y., Armando A.M., Quehenberger O., Yan C., Dennis E.A. (2014). Comprehensive ultra-performance liquid chromatographic separation and mass spectrometric analysis of eicosanoid metabolites in human samples. J. Chromatogr. A.

[166] Watrous J.D., Niiranen T.J., Lagerborg K.A., Henglin M., Xu Y.J., Rong J., Sharma S., Vasan R.S., Larson M.G., Armando A. (2019). Directed Non-targeted Mass Spectrometry and Chemical Networking for Discovery of Eicosanoids and Related Oxylipins. Cell Chem. Biol..

